# Hierarchical ZnO–Graphite Films Enabling Durable Antifouling and Corrosion Protection of Electrochemical Electrodes in Harsh Wastewater Environments

**DOI:** 10.3390/nano16090547

**Published:** 2026-04-30

**Authors:** Ziqi Chen, Tongyan An, Jianwei Yu

**Affiliations:** 1 School of Water Resources and Environment, China University of Geosciences (Beijing), Beijing 100083, China; cugbczq@163.com; 2 Beijing Municipal Research Institute of Eco-Environmental Protection, Beijing 100037, China; 3 Research Center for Eco-Environmental Sciences, Chinese Academy of Sciences, Beijing 100085, China; jwyu@rcees.ac.cn

**Keywords:** hierarchical films, ZnO–graphite composites, antifouling interfaces, corrosion protection, electrochemical electrodes, harsh wastewater

## Abstract

In microbial electrochemical coupled treatment technology, the performance of electrodes critically affects the overall efficiency of wastewater treatment systems. Electrochemical electrodes in harsh wastewater often fail due to coupled organic fouling and corrosion. Herein, hierarchical ZnO–graphite composite films are developed as durable active interfaces. Fabricated via scalable spraying, the films feature coral-like architectures composed of ZnO nanoparticles interconnected by a conductive graphite network. Characterization confirms uniform elemental integration and preserved ZnO crystallinity. The films exhibit strong hydrophilicity, facilitating a stable hydration layer for effective underwater oleophobicity. Crucially, electrochemical tests in aggressive simulated landfill leachate demonstrate significant corrosion suppression and fouling resistance. Simultaneously, the embedded graphite phase ensures stable electrical conductivity (<5% variation) over prolonged immersion. This work establishes a robust interfacial design strategy for durable electrochemical sensors in complex wastewater environments.

## 1. Introduction

Ensuring the long-term reliability of electrochemical sensors within wastewater treatment infrastructure represents a significant technological bottleneck, particularly for applications involving chemically aggressive matrices like industrial effluents and landfill leachates [1,2]. Such environments are characterized by severe chemical conditions, including high chloride concentrations (often exceeding 3000 mg L^−1^), substantial organic loads (chemical oxygen demand, COD > 2000 mg L^−1^), and complex biological constituents, which collectively exert immense stress on functional sensor interfaces [3,4]. While electrochemical sensing remains the method of choice for monitoring critical parameters (e.g., ammonia, nitrate) due to its inherent sensitivity and suitability for in situ deployment [5,6], its practical lifespan in these harsh fluids is notoriously short.

Single treatment processes often fail to achieve satisfactory results for high-concentration organic wastewater. Microbial electrochemical coupled treatment technology combines microorganisms with electrochemistry: a low voltage is applied to the microbial system, and the interactions among the electrochemical electrodes, microorganisms, and substrates effectively stimulate specific microorganisms, enhancing their metabolic activity and thereby improving the pollutant removal efficiency of the reactor.

The performance of the electrodes in the electrochemical system has a critical impact on the reactor’s treatment efficiency. Corrosion of the electrodes by pollutants in high-concentration organic wastewater remains a major challenge for achieving stable treatment performance. The root cause of sensor malfunction is frequently attributed to the synergistic destruction caused by surface fouling and electrochemical corrosion—two degradation pathways that mutually amplify one another in high-salinity wastewater [7]. Specifically, organic foulants (e.g., lipids, humic acids) rapidly accumulate on electrode surfaces, forming insulating barriers that impede electron transfer and distort signal accuracy [8]. Concurrently, the high chloride content aggressively attacks metallic substrates, inducing localized pitting and leading to an irreversible breakdown of electrical continuity [9]. This vicious cycle of signal attenuation and structural disintegration necessitates frequent recalibration or replacement, thereby driving up maintenance costs and hindering the viability of continuous online monitoring.

Current protection methodologies typically suffer from a critical trade-off: they address either fouling or corrosion, but rarely both. Noble-metal coatings (e.g., Pt, IrO_2_) offer superior corrosion resistance yet are prone to rapid deactivation by organic adsorption, alongside being cost-prohibitive for large-scale use [10]. Conversely, conductive polymers like polyaniline can be tailored for antifouling properties, but their stability is often compromised in oxidizing, chloride-rich media, leading to mechanical failure [11]. Alternative strategies utilizing self-assembled monolayers or hydrophilic polymer brushes frequently lack the mechanical robustness required to withstand long-term hydrodynamic shear [12]. Consequently, there remains a critical need for a holistic materials strategy that can simultaneously shield electrodes from fouling and corrosion without severing the electrical connectivity required for sensing.

Nature offers compelling inspiration for solving such multi-objective problems. Hierarchically structured surfaces, resembling the multiscale architectures of coral reefs, have proven effective in managing interfacial interactions in complex saline environments [13,14,15]. Adapting this bio-inspired logic to engineered coatings provides a pathway to independently tune surface morphology, chemical functionality, and bulk properties. Zinc oxide (ZnO), a wide-bandgap semiconductor (~3.3 eV) known for its diverse crystallographic morphologies, serves as a preferred scaffold for constructing such architectures [16,17,18]. Crucially, its surface chemistry can be modified to regulate water and organic interactions, while its rigid framework ensures mechanical durability.

To translate this concept into a functional sensor interface, two key requirements must be balanced. First, surface chemistry must be meticulously engineered: fluorinated groups can lower surface energy to repel oils, while hydrophilic moieties (e.g., carboxylates) induce a tightly bound hydration shell that physically blocks organic adhesion underwater [19,20]. Second, the coating must remain electrically conductive. Since pure oxide films are typically insulating, incorporating a percolating carbonaceous phase, such as nano-graphite, is essential to establish a robust electron transport network within the protective matrix [21].

In this study, we bridge these gaps by developing a hierarchical ZnO–graphite composite film capable of decoupling protection from passivation. Fabricated via a versatile spray-coating technique, the film features a densely interconnected "coral-like" architecture where ZnO provides the structural skeleton and chemical stability, while an embedded graphite network guarantees sustained electrical conductivity. We achieved a dual-regulation of surface wettability by simultaneously introducing fluorinated and hydrophilic ionic groups. Through a systematic investigation covering structural evolution, wetting dynamics, corrosion behavior, and long-term electrical stability, we demonstrate that this integrated design effectively resolves the conflict between durability and sensitivity. This work provides a robust, scalable solution for extending the service life of electrochemical electrodes in many demanding wastewater environments.

## 2. Materials and Methods

### 2.1. Materials and Fabrication of Hierarchical ZnO–Graphite Films 

Chemical reagents used in this study were of analytical grade and utilized directly without additional purification. Commercially available zinc oxide nanoparticles (ZnO, 99.8%, 30 ± 10 nm) and nano-graphite powder (99.5%, <100 nm) were acquired from Sigma-Aldrich. Perfluorobutanoic acid (PFBA, 97%), sodium hydroxide (NaOH, 98%), absolute ethanol, and other essential chemicals were sourced from Sinopharm Chemical Reagent Co., Ltd. Invar alloy sheets (Fe–36Ni, 10 × 10 × 1 mm^3^;) served as representative metallic substrates to simulate electrochemical electrodes operating in harsh wastewater environments. Invar alloy was chosen because its low thermal expansion coefficient matches the ZnO–graphite coating, minimizing thermal stress, and its moderate intrinsic corrosion resistance enables a clear evaluation of the coating’s protective performance.

Composite suspensions containing varying ZnO loadings (0.02, 0.04, 0.06, and 0.08 g mL^−1^) were formulated using a stepwise surface modification and dispersion technique. The detailed fabrication procedure, specifically regarding PFBA modification and spray parameters, was optimized based on preliminary internal trials in our laboratory. Briefly, PFBA (3.0 g) was dissolved in absolute ethanol (50 mL) under magnetic stirring (500 rpm) for 30 min. Subsequently, NaOH (0.15 g) was introduced to generate perfluorobutanoate species, facilitating the in situ formation of surface-active fluorinated anions. Predetermined amounts of ZnO nanoparticles (1, 2, 3, and 4 g) together with nano-graphite (0.5, 1.0, 1.5, and 2.0 g, respectively) were then incorporated into the solution. The resulting mixtures were kept at 60 °C and stirred for 4 h to promote surface functionalization of ZnO particles and homogeneous dispersion of the conductive graphite phase.

Prior to deposition, Invar substrates were ultrasonically cleaned in ethanol for 20 min, dried under nitrogen flow, and coated with a thin conductive silver paste interlayer to enhance electrical contact and interfacial adhesion. The prepared suspensions were deposited using an airbrush equipped with a 0.3 mm nozzle. Spraying was performed at a nozzle-to-substrate distance of 15 cm under a constant air pressure of 1.4 bar (20 psi) for 10 s. Following a 5-min leveling period at room temperature, the as-sprayed films were thermally cured at 150 °C for 30 min in a programmable oven and subsequently allowed to cool naturally. The resulting samples were denoted as ZG-1, ZG-2, ZG-3, and ZG-4 according to increasing ZnO content.

### 2.2. Structural and Chemical Characterization

The surface morphology and microstructure of the composite films were investigated via field-emission scanning electron microscopy (FE-SEM, Hitachi SU8020) with an accelerating voltage set to 5 kV. To assess the chemical uniformity of the ZnO and graphite components, elemental composition and spatial distribution were mapped using energy-dispersive X-ray spectroscopy (EDS, Oxford X-MaxN) coupled to the SEM system. The phase composition and crystal structure were determined by X-ray diffraction (XRD, Bruker D8 Advance) using Cu Kα radiation (λ = 1.5418 Å) across a 2θ range of 10–80° at a scan speed of 2°·min^−1^. This analysis was employed to verify the crystallinity of ZnO and to confirm that the spray-coating and thermal curing processes did not induce phase transitions in the constituent materials.

The surface functional groups and chemical bonding were probed using Fourier-transform infrared spectroscopy (FTIR, Nicolet iS50) in attenuated total reflectance (ATR) mode. Spectra were acquired over the range of 4000–500 cm^−1^ with a resolution of 4 cm^−1^ by averaging 32 scans. Furthermore, surface elemental states were analyzed via X-ray photoelectron spectroscopy (XPS, Thermo Scientific K-Alpha) using monochromatic Al Kα radiation (1486.6 eV). Survey and high-resolution spectra were recorded with pass energies of 100 eV and 20 eV, respectively, and binding energies were calibrated against the C 1s peak at 284.8 eV.

### 2.3. Performance Evaluation

Surface wettability was evaluated using an optical contact-angle goniometer (Dataphysics OCA25). Static contact angles of deionized water and diiodomethane were measured at ambient conditions (25 ± 1 °C, 50 ± 5% relative humidity (RH)) using 3 μL droplets. Each reported value represents the average of five measurements taken from distinct locations on the surface.

Antifouling behavior was assessed under both ambient exposure and simulated harsh wastewater conditions. For natural environment testing, samples were stored under laboratory atmosphere for 15 days, during which contact-angle measurements were recorded daily. Accelerated antifouling tests were conducted by immersing samples in simulated wastewater containing 3.5 wt% NaCl and organic contaminants (oil–water volume ratio of 1:3) at 50 ± 1°C for 120 min. The accelerated antifouling and immersion tests were conducted at 50 ± 1 °C to simulate the elevated temperatures often encountered in real landfill leachate and industrial effluents (e.g., during summer or after biological treatment), while also providing a mild acceleration factor for evaluating long-term stability within a practical timeframe. Contact angles were measured at 30-min intervals to monitor surface wettability evolution.

To gauge long-term stability in aggressive media, additional prolonged immersion tests were performed. Coated and uncoated samples were continuously immersed in simulated harsh wastewater (3.5 wt% NaCl with organic contaminants) at 50 ± 1 °C for extended durations of 24 h and 72 h. After each immersion period, samples were gently rinsed with deionized water, dried under nitrogen flow, and subjected to electrochemical and wettability measurements to assess the retention of protective performance. 

Coating adhesion was quantified by progressive-load nanoindentation using a Berkovich diamond indenter (Anton Paar NST^3^). The critical load corresponding to interfacial delamination was determined from five independent measurements for each sample. 

Electrochemical corrosion behavior was investigated using a Gamry Interface 1010E potentiostat in a three-electrode configuration with a simulated wastewater electrolyte (3.5 wt% NaCl). 

Potentiodynamic polarization measurements were performed after a stabilization period of 30 min, scanning from −0.5 V to +0.3 V versus open-circuit potential (OCP) at a rate of 1 mV s^−1^. Corrosion potential (*E*_corr_) was obtained from the polarization curves. For passive systems, the passive current density (*j*_pass_) was taken at a potential of OCP+200 mV (within the passive region) as a measure of the corrosion rate. Electrochemical impedance spectroscopy (EIS) measurements were conducted at open-circuit potential over a frequency range from 10^5^ to 10^−2^ Hz with an AC amplitude of 10 mV. 

To evaluate the durability of the electrodes under conditions representative of aggressive real-world wastewater, a simulated landfill leachate electrolyte was prepared according to typical compositions reported in Ref. [22]. The solution consisted of 3.5 wt% NaCl to simulate a high-salinity environment, 800 mg L^−1^ NH_4_Cl (calculated as NH_3_–N) to represent high concentrations of ammonium nitrogen, and 1500 mg L^−1^ sodium acetate to simulate a high COD organic load. The pH of the electrolyte was adjusted to 8.0 ± 0.2. All electrochemical and long-term immersion tests using this simulated leachate were conducted at a controlled temperature of 50 ± 1 °C to assess the coating stability under accelerated degradation conditions.

To simulate continuous electrochemical stress relevant to sensor operation in harsh wastewater environments, chronoamperometry tests were additionally performed. The electrodes were polarized at a constant potential of OCP + 200 mV to simulate mild anodic polarization conditions relevant to electrochemical sensor operation for 2 h in simulated wastewater, and the transient current response was recorded to evaluate the stability of the coating under sustained electrochemical bias. Bare Invar and ZG-3 coated electrodes were tested for direct comparison.

Electrical conductivity was evaluated through sheet-resistance measurements using a four-point probe system (Keithley 2450 SourceMeter). For each sample, five measurements were performed to ensure statistical reliability.

## 3. Results and Discussion

### 3.1. Morphological and Structural Evolution of Hierarchical ZnO–Graphite Films 

**Figure 1:**
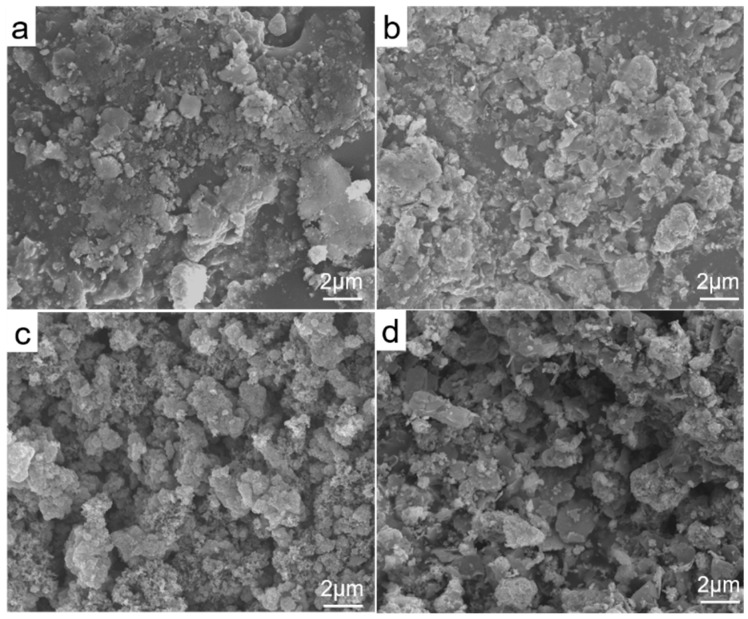
SEM images illustrating the concentration-dependent evolution of hierarchical ZnO–graphite thin films deposited by spray coating: (**a**) ZG-1 (0.02 g mL^−1^), (**b**) ZG-2 (0.04 g mL^−1^), (**c**) ZG-3 (0.06 g mL^−1^), and (**d**) ZG-4 (0.08 g mL^−1^).

The morphological evolution of the ZnO–graphite composite films as a function of ZnO concentration is shown in Figure 1. At a low ZnO concentration of 0.02 g mL^−1^ (ZG-1), the deposited film exhibits a heterogeneous and loosely packed morphology dominated by irregular nano-graphite flakes, with ZnO nanoparticles sparsely distributed on the surface (Figure 1a). The insufficient ZnO loading leads to incomplete surface coverage and limited interparticle connectivity, resulting in a mechanically and structurally discontinuous film. 

When the ZnO concentration is increased to 0.04 g mL^−1^ (ZG-2), ZnO nanoparticles begin to form interconnected clusters that partially encapsulate the graphite flakes (Figure 1b). This intermediate morphology reflects the onset of multiscale structural organization, in which nanoscale ZnO building blocks coexist with microscale aggregated features. However, the overall structure remains relatively open, with a non-uniform pore distribution.

An optimal hierarchical morphology is obtained at a ZnO concentration of 0.06 g mL^−1^ (ZG-3). As shown in Figure 1c, densely packed ZnO nanospheres with diameters in the range of 50–80 nm assemble into continuous microscale aggregates, forming an interconnected porous network across the film surface. Elemental mapping results (Figure S1) further confirm that the nano-graphite phase is homogeneously embedded within the ZnO framework rather than simply coating the surface, thereby contributing to both structural integrity and electrical connectivity. The resulting architecture exhibits multilevel surface features composed of nanoscale roughness superimposed on microscale branched domains, which bear a qualitative resemblance to coral-inspired hierarchical organizations reported in natural and engineered systems [23,24]. Importantly, this “coral-like” description is used here as a structural analogy rather than a biomimetic claim, emphasizing the presence of a quantified hierarchical porous network. This hierarchical architecture may play an important role in regulating subsequent interfacial wettability and electrochemical behavior, as discussed in Section 3.3 and Section 3.4.

Further increasing the ZnO concentration to 0.08 g mL^−1^ (ZG-4) leads to a deterioration of the hierarchical organization (Figure 1d). Excessive ZnO loading promotes nanoparticle agglomeration and densification, resulting in reduced pore connectivity and partial surface segregation of the graphite component. The collapse of the multiscale architecture at high concentration highlights the existence of a narrow processing window for achieving stable hierarchical structures during spray deposition, underscoring the critical role of ZnO concentration in governing film morphology. 

### 3.2. Surface Chemistry and Modification Verification

**Figure 2:**
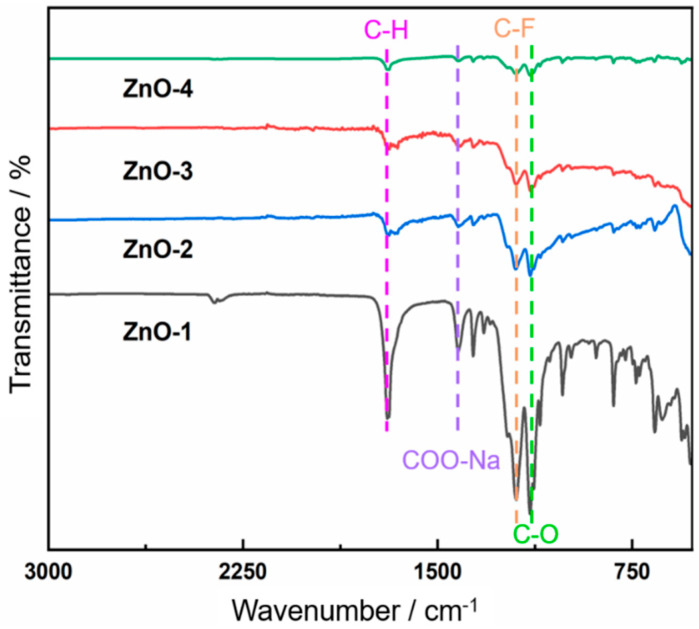
FTIR spectra of hierarchical ZnO–graphite composite films with different ZnO concentrations, showing the coexistence of fluorinated (C–F) and ionic (COO^−^Na^+^) surface functional groups.

Fourier-transform infrared (FTIR) spectroscopy was employed to examine the surface functional groups of the ZnO–graphite composite films (Figure 2). Across all ZnO concentrations, characteristic absorption bands located at approximately 1196 cm^−1^ and 1420 cm^−1^ were consistently observed, corresponding to C–F stretching vibrations and COO^−^Na^+^ asymmetric stretching modes, respectively [25,26]. The simultaneous presence of these bands indicates the coexistence of fluorinated moieties and ionic hydrophilic groups on the film surface. The relative intensity of the C–F and COO^−^Na^+^ bands exhibited concentration-dependent variations, with the ZG-3 sample showing a comparatively balanced spectral contribution from both functional groups. 

To quantitatively assess this balance, we integrated the peak areas of the C–F band (~1196 cm^−1^) and the COO^−^Na^+^ band (~1420 cm^−1^). The calculated peak area ratios (C–F/COO^−^Na^+^) are: ZG-1: 2.8, ZG-2: 1.6, ZG-3: 1.1, ZG-4: 0.6. A ratio close to 1 (ZG-3) indicates a nearly equimolar surface population of fluorinated (low-surface-energy) and ionic hydrophilic (hydration-inducing) groups. This balance is critical because the fluorinated groups provide air-phase oleophobicity, while the carboxylate groups promote a stable hydration layer under water. Consequently, ZG-3 achieves both effective fouling resistance and corrosion protection. 

**Figure 3:**
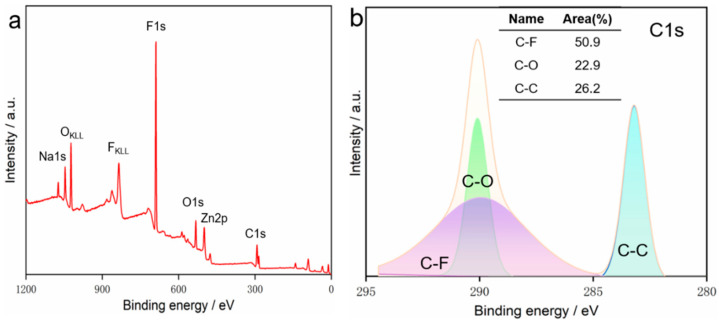
XPS spectra of the ZG-3 hierarchical ZnO–graphite film: (**a**) survey spectrum and (**b**) high-resolution C 1s spectrum deconvoluted into C–F, C–O, and C–C components.

X-ray photoelectron spectroscopy (XPS) was used to probe the surface chemical states of the composite films, with ZG-3 selected as a representative sample (Figure 3). The survey spectrum confirmed the presence of Zn, O, C, F, and Na elements, consistent with the designed composite composition. High-resolution C 1s spectra could be deconvoluted into three distinct components centered at 290.3 eV (C–F, 50.9%), 289.7 eV (C–O, 22.9%), and 283.3 eV (C–C, 26.2%). The relative contributions of these components reflect the surface-sensitive nature of XPS measurements rather than bulk composition, yet they provide clear evidence for the successful incorporation of fluorinated species together with oxygen-containing functional groups.

Elemental distribution was further examined by energy-dispersive X-ray spectroscopy (EDS) mapping (Figure S1). The uniform spatial distribution of F and Na signals throughout the hierarchical ZnO–graphite framework supports the co-localization of hydrophobic and hydrophilic functionalities within the composite film, rather than phase-separated surface domains. Combined with the structural information discussed in Section 3.1, these results confirm that the surface modification strategy is compatible with the formation of the hierarchical architecture. 

### 3.3. Wettability and Anti-Fouling Performance

**Figure 4:**
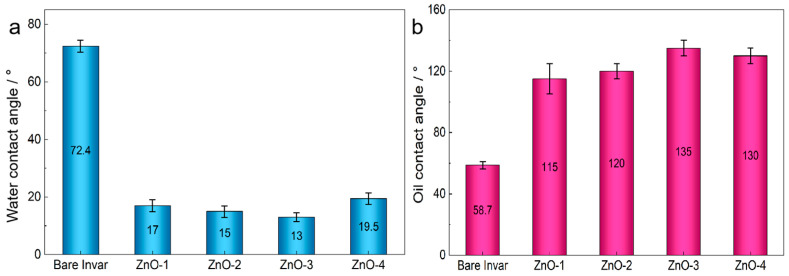
Static contact-angle measurements of the ZnO–graphite composite films: (**a**) water contact angles and (**b**) diiodomethane contact angles for bare Invar and ZG-1 to ZG-4.

The surface wettability of the ZnO–graphite composite films was systematically evaluated by static contact-angle measurements using deionized water and diiodomethane as probe liquids (Figure 4). As shown in Figure 4a, the water contact angle of the pristine Invar substrate was 72.4° ± 2.1°, indicating moderate intrinsic wettability. After coating deposition, all composite films exhibited pronounced hydrophilic behavior, with water contact angles decreasing progressively as the ZnO concentration increased. The ZG-3 film displayed a minimum water contact angle of 12.9° ± 1.8°, reflecting strong surface affinity toward water.

In contrast, diiodomethane contact-angle measurements revealed a clear enhancement in oil repellency upon film formation (Figure 4b). The oil contact angle increased from 58.7° ± 2.4° for bare Invar to a maximum value of 137.1° ± 3.2° for the ZG-3 sample. Although this value does not reach the conventional threshold for superoleophobicity (≥150°), it represents a high degree of oleophobicity under ambient conditions. Accordingly, the term highly oleophobic is used throughout this work to describe the oil-repellent behavior of the composite films.

The apparent coexistence of hydrophilicity and high oleophobicity in air warrants careful interpretation. Diiodomethane was selected as a nonpolar probe liquid commonly used for surface-energy evaluation rather than as a direct surrogate for complex organic foulants in wastewater. The observed wettability behavior is therefore indicative of the surface’s ability to repel low-surface-tension liquids, rather than a direct measure of fouling resistance under aqueous conditions.

**Figure 5:**
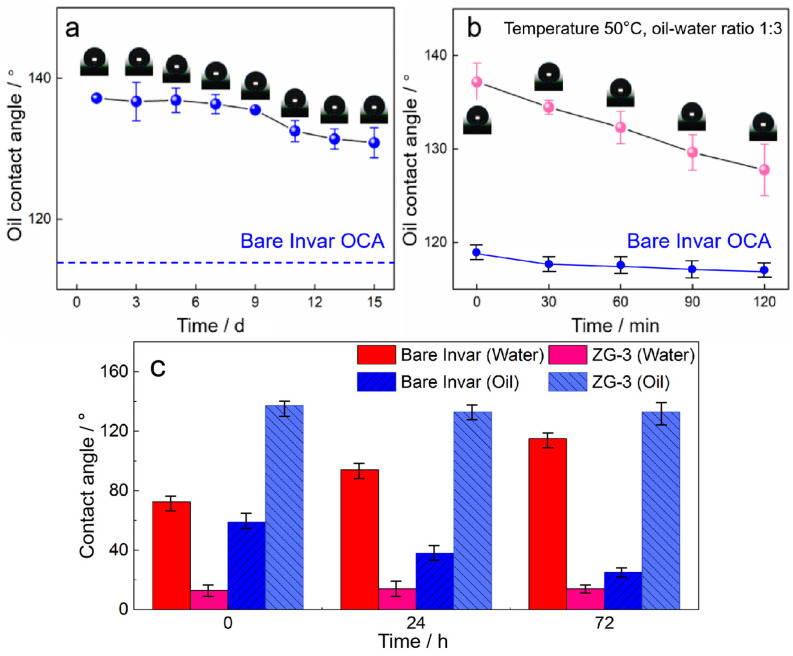
Wettability stability of the bare Invar substrate and the ZG-3 composite film under different fouling conditions: (**a**) natural environment exposure for 15 days (bare Invar shown as horizontal reference lines); (**b**) accelerated testing in simulated harsh wastewater (bare Invar data added as curves); (**c**) Prolonged immersion for 24 h and 72 h in simulated harsh wastewater at 50 °C, showing water contact angle (WCA) and oil contact angle (OCA) for both samples. Error bars represent standard deviations from three independent measurements.

The antifouling performance of the composite films was further evaluated under both ambient exposure and simulated harsh wastewater conditions (Figure 5). During natural environment exposure over 15 days, the ZG-3 film maintained stable water and oil contact angles with minimal variation, suggesting resistance to airborne organic contamination (Figure 5a). In contrast, the bare Invar substrate showed constant but higher water contact angle (~72.4°) and lower oil contact angle (~58.7°) throughout the 15 days, as shown by the horizontal reference lines in Figure 5a. Under accelerated testing in simulated wastewater conditions representative of high-salinity and organic-rich streams, such as landfill leachate and industrial effluents, the wettability of the coated surfaces exhibited only moderate changes over 120 min at 50 °C, whereas the uncoated Invar substrate showed rapid degradation of surface properties (Figure 5b). Specifically, the water contact angle of bare Invar increased sharply from 72.4° to >90° within 60 min and reached >110° after 120 min, while its oil contact angle dropped to ~30°, indicating severe fouling.

To further evaluate antifouling durability under prolonged harsh wastewater exposure, extended immersion tests were conducted at 50 °C for 24 h and 72 h. The results are summarized in Figure 5c. After prolonged immersion, the ZG-3 coating retained a low water contact angle (<15°) and a high oil contact angle with only a minor decrease (<6%), whereas the uncoated Invar surface exhibited pronounced wettability deterioration within the first 24 h. These results confirm that the antifouling behavior of the ZG-3 film is not a transient surface effect but is sustained under prolonged chemical and thermal stress.

For practical wastewater applications, antifouling performance is more appropriately interpreted in terms of underwater oleophobicity induced by water-mediated interfacial interactions rather than air-phase oil repellency. The strong hydrophilicity of the ZG-3 surface facilitates the formation of a tightly bound hydration layer arising from surface carboxylate groups and Zn–OH species. This hydration layer acts as an energetic barrier that inhibits the adhesion of hydrophobic organic foulants when the surface is submerged, a mechanism widely recognized as the basis of underwater oleophobicity and fouling resistance on hydrophilic surfaces [27,28]. Although underwater oil contact-angle measurements were not conducted in this study, the combined wettability data, accelerated fouling tests, and extended immersion stability collectively support the hydration-enabled antifouling function of the hierarchical composite films in aqueous environments. 

Overall, the ZG-3 film demonstrates a favorable balance between hydrophilicity, air-phase oil repellency, and environmental stability. These characteristics are particularly relevant for electrochemical sensors operating in harsh wastewater streams, where prolonged exposure to saline electrolytes, organic foulants, and electrochemical bias demands surfaces with durable antifouling functionality rather than short-lived surface effects.

### 3.4. Corrosion Protection Mechanism 

**Figure 6:**
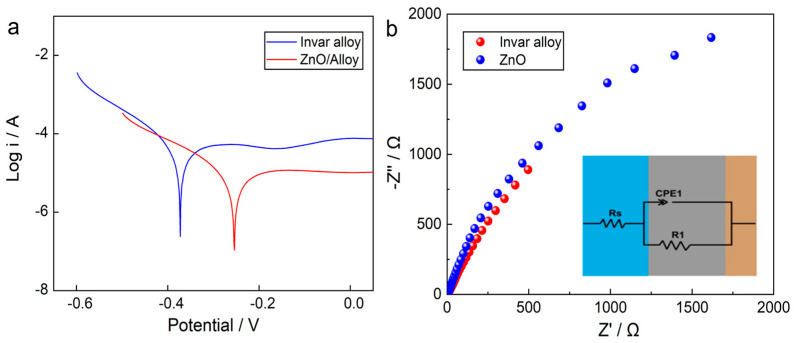
Electrochemical corrosion behavior of bare Invar and ZG-3 coated electrodes in simulated high-chloride wastewater: (**a**) potentiodynamic polarization curves and (**b**) Nyquist plots obtained from electrochemical impedance spectroscopy (EIS) measurements. Inset: equivalent circuit *R*s(CPE1//R_1_), where *R*s = solution resistance, CPE1 = constant phase element (double-layer capacitance), and *R*_1_ = charge-transfer resistance.

Electrochemical measurements demonstrate a pronounced enhancement in corrosion resistance for the hierarchical ZnO–graphite coated electrodes (Figure 6). As shown in Figure 6a, potentiodynamic polarization curves reveal a positive shift in corrosion potential (*E*_corr_) from −0.368 V for bare Invar to −0.229 V for the ZG-3 coated sample, accompanied by a significant reduction in corrosion current density (*j*_pass_) from 2.761 × 10^−5^ to 2.23 × 10^−6^ A·cm^−2^ measured at a potential of OCP+200 mV (within the passive region for both samples). This more than one-order-of-magnitude decrease in corrosion rate indicates effective suppression of electrochemical degradation in simulated high-chloride wastewater.

The enhanced corrosion resistance stems from a robust synergistic mechanism. Primarily, the densely packed hierarchical ZnO framework imposes a physical tortuosity effect. By creating a convoluted diffusion path, the structure significantly delays the penetration of aggressive chloride ions toward the metal substrate, effectively functioning as a permeable barrier that balances protection with electrochemical accessibility. Beyond physical blocking, the intrinsic electronic properties of the crystalline ZnO phase (confirmed by XRD, Figure S2) play a pivotal role. The wurtzite crystal structure of ZnO is a wide-bandgap semiconductor (≈3.3 eV) with native donor defects (oxygen vacancies and zinc interstitials), giving it n-type character. When the coating contacts the electrolyte, a space-charge layer (depletion region) forms at the ZnO/electrolyte interface, raising the energy barrier for electron transfer from the Invar substrate to oxidizing species (e.g., O_2_, Cl^−^). Thus, even without full coverage, crystalline ZnO suppresses anodic dissolution by limiting electron availability for cathodic reactions. This semiconductor-mediated passivation acts synergistically with the physical tortuosity and hydration layer [29].

Further mechanistic insight is provided by electrochemical impedance spectroscopy (Figure 6b). The detailed electrochemical parameters are provided in Table S1 (Supplementary Materials). The high charge-transfer resistance (R_1_ in the equivalent circuit) of ZG-3 (2.8 × 10^5^ Ω·cm^2^) compared to bare Invar (3.2 × 10^3^ Ω·cm^2^) confirms the effective barrier property of the coating. The CPE exponent n for ZG-3 (0.92) is closer to unity than that of bare Invar (0.86), indicating a more homogeneous and defect-poor surface. The Nyquist plots of both bare Invar and ZG-3 coated electrodes exhibit a single depressed semicircle over the measured frequency range, indicating that the corrosion process is predominantly governed by charge-transfer reactions at the electrode–electrolyte interface. Notably, the diameter of the capacitive arc for the ZG-3 coated electrode is significantly larger than that of the bare substrate, reflecting a pronounced increase in charge-transfer resistance (Rct). Equivalent circuit fitting reveals that the Rct value of the coated electrode is approximately two orders of magnitude higher than that of the uncoated Invar substrate. 

The presence of a single, well-defined time constant suggests uniform interfacial behavior without evidence of localized corrosion, coating delamination, or pore-induced electrochemical activity at the initial stage of exposure. This observation indicates that the hierarchical ZnO–graphite coating forms a continuous and defect-limited barrier at the metal–electrolyte interface, effectively suppressing charge-transfer processes associated with corrosion reactions. 

**Figure 7:**
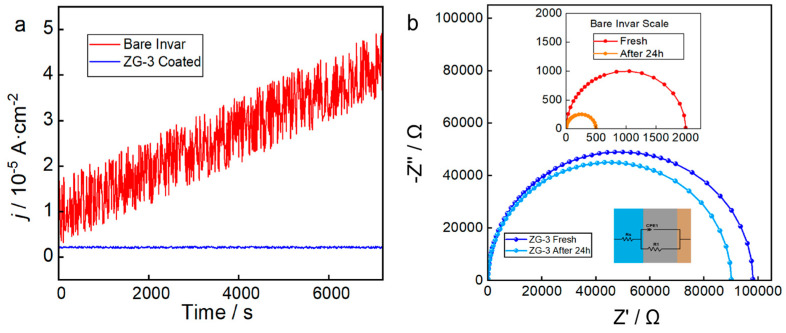
Electrochemical stability of bare Invar and ZG-3 coated electrodes in simulated landfill leachate. (**a**) Chronoamperometric responses (current density, *j*) under constant potential and (**b**) Nyquist plots obtained before and after 24 h immersion.

To further evaluate corrosion stability under conditions more representative of landfill leachate and other harsh wastewater environments, additional electrochemical measurements were conducted in a simulated leachate electrolyte containing high salinity, ammonium species, and organic components (Figure 7). Chronoamperometric responses recorded under a constant applied potential (Figure 7a) show that the bare Invar electrode exhibits a continuously increasing current density, suggesting gradual degradation of the native passive film under the synergistic attack of chloride and ammonium ions, rather than stable pitting corrosion (the polarization curve in Figure 6a does not show a breakdown potential up to OCP + 300 mV) [30,31]. In contrast, the ZG-3 coated electrode maintains a low and stable current response over the entire testing period, demonstrating that the conductive graphite network within the coating does not compromise its barrier properties. Electrochemical impedance spectroscopy measurements collected before and after 24 h immersion in the simulated leachate (Figure 7b) further confirm the durability of the hierarchical coating. While the charge-transfer resistance of the bare substrate decreases markedly after immersion, the ZG-3 coated electrode retains a large capacitive arc with only minor changes in Rct, indicating sustained interfacial stability under prolonged chemical exposure. The same equivalent circuit was used to fit the EIS data in Figure 7b as for Figure 6b. The fitted parameters (including *R*_s_, *R*_ct_, *Q*, and *n*) are provided in Table S1 (Supplementary Materials). Even after 24 h immersion, the charge-transfer resistance (*R*_1_) of ZG-3 remains two orders of magnitude higher than that of bare Invar, and the CPE exponent n stays close to 0.91, confirming the coating’s sustained barrier integrity and surface homogeneity under aggressive leachate conditions. The absence of additional time constants after immersion suggests that the coating does not undergo localized failure or interfacial degradation during extended electrochemical stress.

Collectively, these electrochemical results demonstrate that the hierarchical ZnO–graphite film acts as an effective multifunctional corrosion-protection layer not only in chloride-dominated electrolytes but also under more complex chemical conditions representative of harsh wastewater environments. Rather than relying on a single dominant mechanism, corrosion resistance arises from the coupled effects of physical diffusion barriers, semiconductor-mediated passivation, and hydration-layer-assisted ion transport suppression, enabling durable electrochemical stability under prolonged and chemically aggressive conditions.

### 3.5. Integrated Performance and Structure–Property Relationships

ZG-3 composite film demonstrates a well-balanced combination of mechanical robustness, electrical conductivity, and environmental stability, satisfying key performance requirements for electrochemical sensor protection in harsh wastewater environments. Nanoindentation-based adhesion testing yielded an average critical load of 7.5 ± 0.8 mN (Figure S3), indicating strong interfacial bonding between the coating and the Invar substrate. This level of adhesion is sufficient to withstand hydrodynamic shear stresses commonly encountered in flowing wastewater systems. 

Electrical performance is a critical consideration for protective coatings applied to electrochemical sensors. Four-point probe measurements revealed a sheet resistance of 85 ± 12 Ω·sq^−1^ for the ZG-3 film, confirming the formation of a continuous conductive pathway provided by the embedded nano-graphite network. Importantly, after prolonged immersion in simulated landfill leachate conditions for up to 30 days, the sheet resistance exhibited a variation of less than 5%, indicating that the conductive network remains structurally intact and electrically stable under combined corrosive and fouling stresses. This stability is essential for maintaining signal fidelity in long-term sensing applications. 

**Figure 8:**
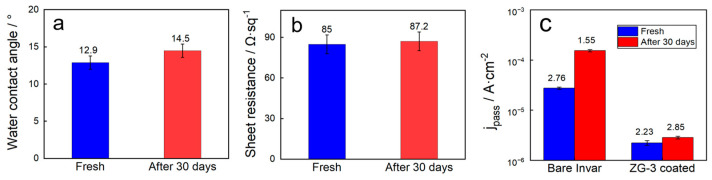
Retention of key interfacial and electrical properties after prolonged immersion (30 days) in simulated landfill leachate. (**a**) Water contact angle (WCA), (**b**) Sheet resistance (four-point probe measurement) of bare Invar and ZG-3—this is distinct from the solution resistance *R*_s_ in the EIS equivalent circuit (Figure 6), and (**c**) corrosion current density measured at OCP + 200 mV (passive region), corresponding to *j*_pass_. Specific values: Bare Invar (Fresh: WCA= 72.4°, sheet resistance = 12.4 Ω·sq^−1^, *j* = 2.76 × 10^−5^ A·cm^−2^; After 30 days: WCA = 94°, sheet resistance = 15.2 Ω·sq^−1^, *j* = 1.55 × 10^−4^ A·cm^−2^); ZG-3 (Fresh: WCA = 12.9°, sheet resistance = 85 Ω·sq^−1^, *j* = 2.23 × 10^−6^ A·cm^−2^; After 30 days: WCA = 14°, sheet resistance = 88 Ω·sq^−1^, *j* = 2.85 × 10^−6^ A·cm^−2^).

The retention of multifunctional properties under prolonged chemical exposure is further summarized in Figure 8. As shown in Figure 8a, the ZG-3 film maintains a low water contact angle after 30 days of continuous immersion in the simulated landfill leachate, indicating preservation of surface hydrophilicity and hydration-layer-forming capability. Figure 8b demonstrates that the sheet resistance (denoted as *R*_sheet_, measured by four-point probe; this is distinct from the solution resistance *R*_s_ in the EIS equivalent circuit) of the coated electrode remains nearly unchanged after immersion, corroborating the long-term electrical stability observed in macroscopic conductivity measurements. In parallel, Figure 8c shows that the corrosion current density of the ZG-3 coated electrode exhibits only minor variation after prolonged exposure, whereas the bare Invar substrate undergoes a pronounced increase in corrosion activity. These results collectively confirm that the hierarchical coating retains its antifouling, conductive, and corrosion-protective functions over extended time scales.

In addition to static conductivity measurements, the electrochemical stability of the conductive network under sustained electrochemical bias was indirectly corroborated by chronoamperometric testing (Section 3.4), where the ZG-3 coated electrodes maintained a low and stable current response over extended polarization periods. This observation indicates that the embedded nano-graphite network remains electrochemically accessible while being effectively shielded from interfacial degradation processes. These results collectively demonstrate that the hierarchical ZnO–graphite architecture decouples electrical functionality from corrosion and fouling pathways.

Beyond individual performance metrics, the hierarchical architecture of the composite film plays a decisive role in enabling multifunctionality. The interconnected porous ZnO framework allows electrolyte access to the electrode surface while simultaneously increasing ion transport tortuosity, thereby reconciling the traditionally conflicting requirements of electrochemical responsiveness and corrosion protection. The hydrophilic surface chemistry further promotes the formation of a stable hydration layer, which facilitates self-cleaning through shear-induced removal of weakly adsorbed foulants under flow conditions. 

Crucially, the sustained antifouling performance (Section 3.3), durable corrosion resistance (Section 3.4), and stable electrical conductivity observed over extended exposure times collectively demonstrate that the protective functionality of the ZG-3 film is governed by intrinsic structure–property relationships rather than transient surface effects. This integrated performance profile is particularly relevant for electrochemical sensors operating under continuous exposure to harsh wastewater environments, where multiple degradation mechanisms act simultaneously over extended time scales.

A comparative analysis of key performance indicators is summarized in Table 1. Compared with conventional noble-metal coatings, conductive polymers, and single-function surface modifications, the ZG-3 film exhibits a more balanced performance profile, particularly in the simultaneous suppression of fouling and corrosion. Such multifunctionality is rarely achieved in single-layer coatings and is especially advantageous in complex wastewater environments where multiple degradation mechanisms operate concurrently.

The concentration-dependent evolution from isolated particles (ZG-1) to an optimally connected hierarchical network (ZG-3), followed by structural overpacking (ZG-4), highlights the critical role of nanoscale assembly in determining macroscopic functionality. This structure–property relationship establishes a direct mechanistic linkage between hierarchical architecture, interfacial chemistry, and long-term functional stability, providing general design guidelines for multifunctional protective coatings beyond the specific material system investigated here. 

### 3.6. Mechanism of Durable Multifunctional Protection in Harsh Wastewater Environments

**Figure 9:**
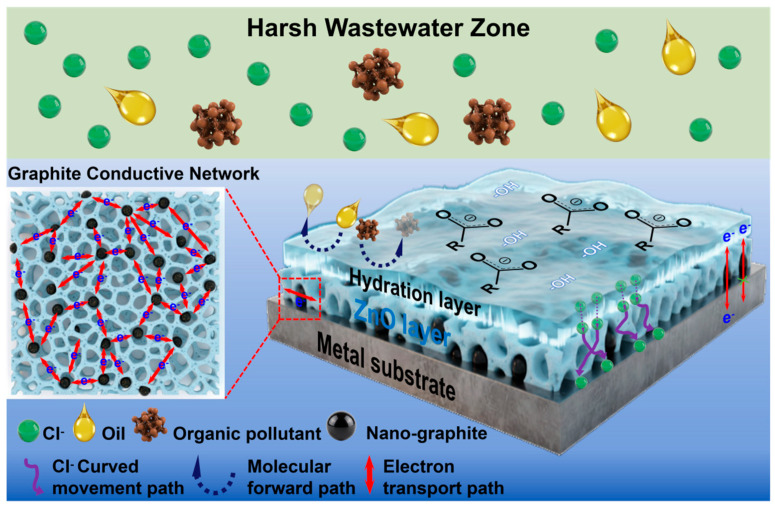
Schematic illustration of the integrated protection mechanism of the hierarchical ZnO–graphite composite film under harsh wastewater conditions.

Figure 9 schematically illustrates the integrated protection mechanism of the hierarchical ZnO–graphite composite film operating in harsh wastewater environments. We attribute the durable multifunctionality of the optimized ZG-3 coating to a cooperative protection mode rather than any single dominant effect. The integration of hierarchical structural regulation, interfacial hydration chemistry, and semiconductor-mediated passivation creates a robust defense system that operates on multiple scales. Specifically, the coral-like architecture physically intercepts foulants and retards ion diffusion, while the hydration layer provides a sub-nanometer shield against organic adhesion. Crucially, this passive protection is underpinned by the embedded graphite network, which ensures that the electrode remains electrochemically "live" for sensing despite the heavy defensive layering. This cooperative coupling allows the system to simultaneously resolve the trade-offs between fouling resistance, corrosion suppression, and electrical conductivity under prolonged stress [32,33].

At the structural level, the coral-like hierarchical ZnO framework forms a three-dimensional interconnected network that significantly increases the tortuosity of ion transport pathways. This geometrical complexity imposes an effective diffusion barrier for aggressive ionic species, particularly chloride ions, without fully blocking electrolyte access to the electrode surface. As demonstrated by the corrosion and impedance results (Section 3.4), this architecture slows down ion migration toward the metal substrate while maintaining the electrochemical accessibility required for sensing. Unlike dense or impermeable barrier coatings, the hierarchical structure reconciles corrosion protection with electrochemical responsiveness, which is critical for electrode operation in high-salinity wastewater environments. 

At the interface, surface chemistry plays a decisive role in regulating fouling and corrosion processes under submerged conditions. The presence of hydrophilic functional groups, including carboxylates and Zn–OH species, promotes the formation of a tightly bound hydration layer at the coating–electrolyte interface. This hydration layer acts as both an energetic and kinetic barrier, suppressing the adsorption of hydrophobic organic foulants and increasing the resistance to ion penetration. The sustained wettability and antifouling stability observed during prolonged immersion tests (Section 3.3) indicate that this hydration-layer-mediated protection is intrinsically stable and structurally supported, rather than relying on transient surface states or weakly bound modifiers.

In parallel, the intrinsic semiconducting properties of ZnO contribute to electrochemical passivation of the underlying metal substrate. The wide bandgap of ZnO limits electron transfer across the coating–electrolyte interface under open-circuit and mild polarization conditions, thereby suppressing anodic dissolution reactions at the Invar surface. The retention of ZnO crystallinity during spray deposition and post-treatment, as confirmed by XRD analysis, is essential for sustaining this passivation effect. This mechanism is reflected in the observed positive shift in corrosion potential, reduced corrosion current density, and elevated charge-transfer resistance (Section 3.4).

Crucially, the embedded nano-graphite phase forms a continuous percolating conductive network throughout the hierarchical ZnO matrix. This network provides stable electron transport pathways that preserve electrochemical functionality, even as the ZnO framework and hydration layer regulate ionic transport and interfacial reactions. The stable sheet resistance after long-term exposure to harsh wastewater and the sustained current response during chronoamperometric testing (Section 3.5) demonstrate that electrical conductivity is effectively decoupled from interfacial degradation processes, enabling the coating to function as an electrochemically active interface rather than a passive protective barrier.

Overall, the durable multifunctional protection of the ZG-3 film arises from the cooperative action of hierarchical structural barriers, hydration-layer-dominated interfacial regulation, semiconductor passivation, and conductive network reinforcement. This integrated mechanism accounts for the simultaneous enhancement of antifouling performance, corrosion resistance, and electrical stability under conditions representative of landfill leachate and industrial effluents containing complex mixtures of salts and organics. Importantly, the protection strategy is governed by intrinsic structure–property relationships rather than short-lived surface modifications, providing a robust and application-relevant design paradigm for electrochemical sensors operating in harsh and chemically complex wastewater environments.

Hierarchical ZnO architecture increases ion diffusion tortuosity, hydrophilic functional groups generate a stable hydration layer that suppresses organic fouling and ion transport, ZnO provides semiconductor passivation against electrochemical corrosion, and the embedded nano-graphite network maintains electrical conductivity. The coupled mechanisms enable durable antifouling, corrosion resistance, and electrochemical stability during prolonged exposure to high-salinity and organic-rich wastewater. 

## 4. Conclusions and Outlook

This work reports the development of a hierarchical ZnO–graphite composite thin film fabricated via a facile and scalable spray-coating process, aimed at improving the operational durability of electrochemical electrodes in harsh wastewater environments. By systematically tuning the ZnO nanoparticle concentration, the structural, chemical, and functional characteristics of the composite films were optimized, with the ZG-3 formulation (0.06 g·mL^−1^) exhibiting a well-defined hierarchical architecture, homogeneous elemental distribution, and a favorable balance of interfacial properties. 

The optimized ZG-3 film demonstrates a combination of strong hydrophilicity and air-phase oil repellency, together with stable antifouling behavior under both ambient exposure and simulated landfill leachate conditions containing high concentrations of ammonia nitrogen and organic contaminants. Rather than relying on extreme wetting states, the antifouling performance is primarily associated with hydration-layer-mediated interfacial interactions, which are particularly relevant under submerged operating conditions. 

Electrochemical measurements further reveal that the hierarchical coating significantly suppresses chloride-induced corrosion, as evidenced by a positive shift in corrosion potential and a marked reduction in corrosion current density compared with bare Invar substrates. Crucially, the coating maintains its protective efficacy and structural integrity even under accelerated electrochemical stress in aggressive leachate electrolytes.

In addition to interfacial protection, the composite film maintains mechanical robustness and electrical functionality. The incorporation of a percolating nano-graphite network enables continuous electrical pathways that remain stable over 30 days of immersion, with sheet resistance variations of less than 5%. This effectively addresses the commonly conflicting requirements of corrosion resistance and electrochemical accessibility in sensor protection layers.

The overall performance of the coating arises from the synergistic integration of multiple structure–property relationships, including (i) a hierarchical porous architecture that increases ion transport tortuosity, (ii) chemically heterogeneous yet spatially co-localized surface functional groups that promote interfacial hydration, (iii) semiconductor-mediated passivation provided by crystalline ZnO, and (iv) a conductive carbon network that stabilizes charge transport. This integrated design framework enables the simultaneous mitigation of fouling and corrosion while retaining electrochemical functionality, which remains challenging for conventional single-function coatings.

From an application perspective, the proposed ZnO–graphite thin-film system provides a scalable and materials-efficient protection strategy for electrochemical electrodes deployed in chemically aggressive wastewater streams, such as landfill leachates and industrial effluents characterized by high salinity and organic loading. Although demonstrated on Invar alloy as a representative engineering substrate, the coating concept is not restricted to a specific electrode material and may be extended to other metallic or conductive substrates commonly used in environmental electrochemical systems. 

Microbial electrochemical coupled treatment technology combines the low cost of biological methods with the high efficiency of chemical methods. Developing applicable microbial electrochemical coupled treatment technologies for high-concentration organic wastewater, such as landfill leachate, can reduce costs and improve the overall efficiency of wastewater treatment systems.

Electrode fabrication is a crucial step in microbial electrochemical coupled treatment technology. The material type, resistance to fouling and corrosion, and the durability of the surface structure directly affect the overall performance of the reactor. Therefore, research on electrode types and performance enhancement will provide support for the application of microbial electrochemical coupled treatment technology in a wider range of scenarios.

Future studies will focus on the use of environmentally benign surface modifiers, extended durability evaluation under real-world field conditions, and integration with functional sensing platforms. Beyond wastewater monitoring, the underlying design principles outlined in this work may also be applicable to other electrochemical systems requiring multifunctional surface protection, including marine sensing and bioelectronic interfaces.

## Figures and Tables

**Table 1:** Comparison of key performance metrics of the ZG-3 hierarchical ZnO–graphite film with representative protective strategies for electrochemical sensors operating in harsh wastewater environments.

Protective Strategy	Antifouling Performance	*Corrosion Resistance	Electrical Conductivity	Mechanical Robustness	Long-Term Stability	Representative Limitations
Noble-metal coatings (Pt, IrO_2_) [10]	Moderate	Excellent	Excellent	Good	Excellent	High cost; susceptible to organic fouling
Conductive polymers (PANI, PEDOT:PSS) [11]	Good	Moderate	Good	Moderate	Limited	Chemical degradation in saline/oxidizing media
*Hydrophilic polymer coatings [12]	Good	Poor–moderate	Poor	Poor	Limited	Insufficient corrosion protection; weak adhesion
Superhydrophobic coatings [13]	Excellent (air)	Poor	Poor	Poor	Limited	Loss of function under immersion; low durability
ZG-3 hierarchical ZnO–graphite film (this work)	Good (hydration-layer mediated)	Excellent	Good (85 ± 12 Ω·sq^−1^)	Good (7.5 ± 0.8 N)	Excellent (>30 days, <5% change)	Performance dependent on structural optimization

Note: *Corrosion resistance (high Cl^−^); *Hydrophilic polymer coatings (PEG, zwitterionic layers); PANI (polyaniline), PEDOT:PSS (poly(3,4-ethylenedioxythiophene):poly(styrene sulfonate)), PEG (polyethylene glycol).

## Data Availability

The data presented in this study are available on request from the corresponding author.

## Supplementary Materials

The following supporting information can be downloaded at: https://www.mdpi.com/article/10.3390/nano16090547/s1, Figure S1: SEM image and corresponding EDS elemental mappings of the ZG-3 film: (a) SEM image of the representative hierarchical region selected for analysis; elemental maps of (b) carbon (C), (c) oxygen (O), (d) zinc (Zn), (e) fluorine (F), and (f) sodium (Na). The homogeneous spatial distribution of F and Na indicates the uniform incorporation of fluorinated and ionic surface-modifying species throughout the hierarchical ZnO–graphite framework; Figure S2: X-ray diffraction (XRD) patterns of the ZnO–graphite composite films (ZG-1 to ZG-4). Diffraction peaks corresponding to hexagonal wurtzite ZnO (JCPDS No. 36-1451), graphite (C, JCPDS No. 75-1621), and the Invar alloy substrate are identified. The results confirm that the spray deposition and surface modification processes preserve the crystalline structure of ZnO; Figure S3: Coating adhesion strength measurement. Bar chart showing the binding force values obtained from five independent nano-scratch tests on the ZG-3 film. The average adhesion strength is calculated to be 7.5 ± 0.8 mN, demonstrating robust interfacial bonding between the film and the Invar alloy substrate, which is critical for withstanding mechanical shear in flowing wastewater. Table S1: Electrochemical corrosion parameters derived from potentiodynamic polarization and EIS (Figure 6 and Figure 7) for bare Invar and ZG-3 coated electrode in simulated high-chloride wastewater (fresh) and after 24 h immersion in simulated landfill leachate.
