# Supplementary material for: Hierarchical ZnO–Graphite Films Enabling Durable Antifouling and Corrosion Protection of Electrochemical Electrodes in Harsh Wastewater Environments
Source: Nanomaterials (Basel). 2026 Apr 30;16(9):547. doi: 10.3390/nano16090547 (PMC13164925; doi:10.3390/nano16090547)
Supplement: Supplementary file 1 [file nanomaterials-16-00547-s001.zip › nanomaterials-4262648-supplementary.pdf]

Supplementary Materials

Hierarchical ZnO–Graphite Films Enabling Durable Antifouling and Corrosion Protection of Electrochemical Electrodes in Harsh Wastewater Environments

Ziqi Chen [1], Tongyan An [2,*] and Jianwei Yu [3]

1 School of Water Resources and Environment, China University of Geosciences (Beijing), Beijing 100083, China; cugbczq@163.com
2 Beijing Municipal Research Institute of Eco-Environmental Protection, Beijing 100037, China
3 Research Center for Eco-Environmental Sciences, Chinese Academy of Sciences, Beijing 100085, China; jwyu@rcees.ac.cn
* Correspondence: antongyan@cee.cn

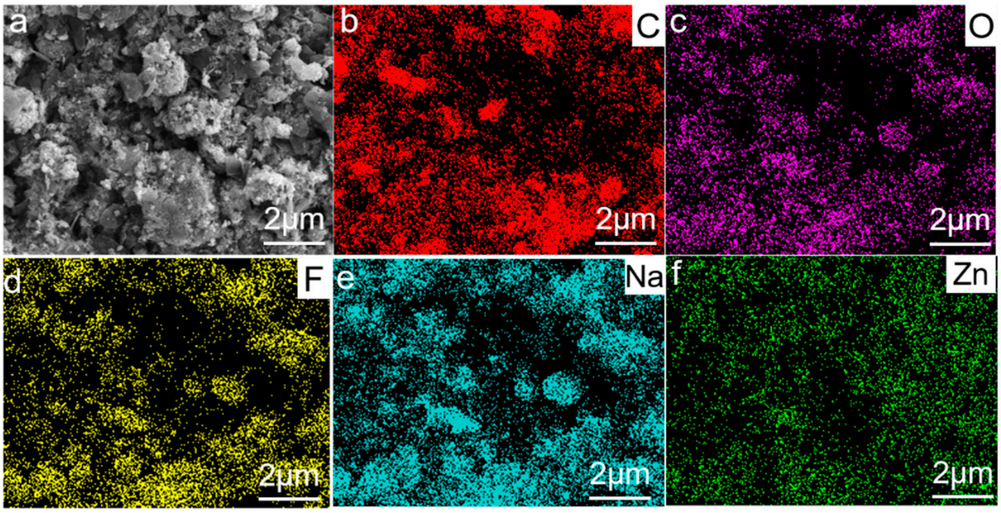

Figure S1. SEM image and corresponding EDS elemental mappings of the ZG-3 film: (a) SEM image of the representative hierarchical region selected for analysis; elemental maps of (b) carbon (C), (c) oxygen (O), (d) fluorine (F), (e) sodium (Na), and (f) zinc (Zn). The homogeneous spatial distribution of F and Na indicates the uniform incorporation of fluorinated and ionic surface-modifying species throughout the hierarchical ZnO–graphite framework.

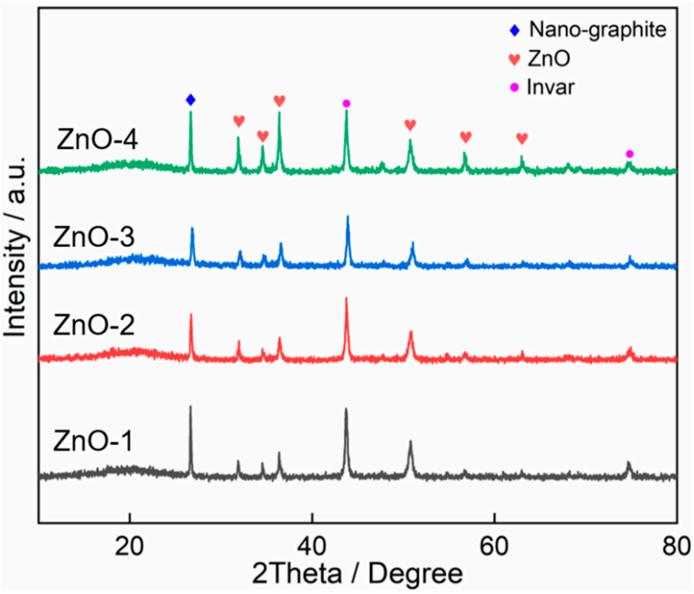

Figure S2. X-ray diffraction (XRD) patterns of the ZnO–graphite composite films (ZG-1 to ZG-4). Diffraction peaks corresponding to hexagonal wurtzite ZnO (JCPDS No. 36-1451), graphite (C, JCPDS No. 75-1621), and the Invar alloy substrate are identified. The results confirm that the spray deposition and surface modification processes preserve the crystalline structure of ZnO.

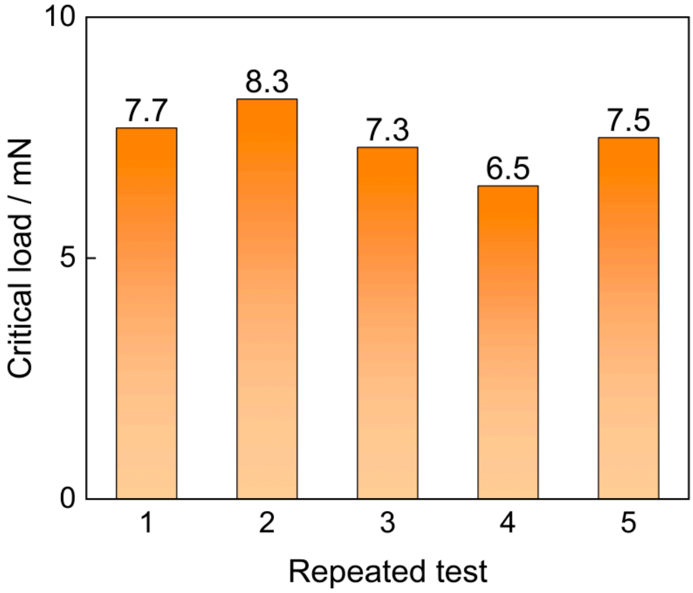

Figure S3. Coating adhesion strength measurement. Bar chart showing the binding force values obtained from five independent nano-scratch tests on the ZG-3 film. The average adhesion strength is calculated to be 7.5±0.8 mN, demonstrating robust interfacial bonding between the film and the Invar alloy substrate, which is critical for withstanding mechanical shear in flowing wastewater.

Table S1. Electrochemical corrosion parameters derived from potentiodynamic polarization and EIS (Figures 6 and 7) for bare Invar and ZG-3 coated electrode in simulated high-chloride wastewater (fresh) and after 24 h immersion in simulated landfill leachate.

**Parameter**	**Symbol (unit)**	**Bare Invar (fresh)**	**ZG-3 (fresh)**	**Bare Invar (24 h leachate)**	**ZG-3 (24 h leachate)**
Corrosion potential	E_{corr} (V vs. OCP)	−0.368	−0.229	−0.401	-0.241
Passive current density[1]	j_{pass} (A·cm^{-2})	2.76×10^{-5}	2.23×10^{-6}	3.15×10^{-5}	2.41×10^{-6}
Anodic Tafel slope	b_a (mV·dec^{-1})	85.3	112.6	88.1	108.9
Cathodic Tafel slope	b_c (mV·dec^{-1})	−78.9	−105.4	−81.2	−102.3
Inhibition efficiency[2]	*IE* (%)	-	91.9	-	92.3
Solution resistance[3]	R_s (Ω·cm^2)	12.4	13.1	13.2	13.5
Charge-transfer resistance	R_{ct} (Ω·cm^2)	3.2×10^3	2.8×10^5	2.9×10^3	2.5×10^5
CPE constant[3]	Q (Ω^{-1}·cm^{-2}·s^n)	2.1×10^{-4}	1.6×10^{-5}	2.3×10^{-4}	1.8×10^{-5}
CPE exponent	n	0.86	0.92	0.85	0.91

Note: 1. For bare Invar (which exhibits a stable passive state), j_{pass} is measured at a potential of OCP+200 mV (within the passive region) instead of using Tafel extrapolation. The same potential is used for ZG-3 for comparison.

2. IE=(1−$j_{pass,coated}$/$j_{pass, bare}$)×100%.

3. These parameters correspond to the equivalent circuit shown in Figure 6b: R_s-solution resistance (labeled Rs in the figure), R_{ct} = charge-transfer resistance (labeled R_1), and Q= constant phase element constant (labeled CPT1). The CPE exponent n is dimensionless.
